# Management of Cardiovascular Disease Risk in Rheumatoid Arthritis

**DOI:** 10.3390/jcm11123487

**Published:** 2022-06-17

**Authors:** Patrick H. Dessein, Miguel A. Gonzalez-Gay

**Affiliations:** 1 Departments of Medicine and Physiology, Faculty of Health Sciences, Universtiy of Witwatersrand, Johannesburg 2000, South Africa; 2 Division of Rheumatology and Epidemiology, Genetics and Atherosclerosis Research Group on Systemic Inflammatory Diseases, Hospital Universitario Marques de Valdecilla, Instituto de Investigacion Marques de Valdecilla (IDIVAL), 39011 Santander, Spain; miguelaggay@hotmail.com; 3 Department of Medicine and Psychiatry, Universidad de Cantabria, 39011 Santander, Spain

Cardiovascular diseases, including ischemic heart disease and stroke, reportedly comprise the top two causes of global mortality [1]. Eighty percent of cardiovascular events are preventable in individuals younger than 75 years old [2]. The prevention of cardiovascular events with antihypertensive and lipid-lowering agents in the general population effectively reduces costs [3]. Importantly in the present context, in patients with rheumatoid arthritis (RA), a ~60% increase in cardiovascular mortality is observed [4]. A recent meta-analysis confirmed that the risk of myocardial infarction and stroke is also increased by ~50% and ~20%, respectively, in spondyloarthritis patients, including those with ankylosing spondylitis (AS) and psoriatic arthritis (PsA) [5]. Reported evidence indicates that cardiovascular risk in individuals with inflammatory joint diseases is mediated by adverse traditional cardiovascular risk factor profiles, as well as systemic inflammation and their interactions and genetic factors [6]. Additionally, medications used to treat disease activity [7] and population origin [8] both impact cardiovascular risk in individuals with inflammatory joint diseases. The enhanced atherogenesis that is reported to occur in individuals with inflammatory joint diseases awaits further elucidation [6,8,9]. Cardiovascular disease risk assessment and prevention are currently suboptimal in individuals with inflammatory joint diseases [6,8,9].

This Special Issue provides six high-quality studies that can improve our understanding of cardiovascular risk and its management in patients with inflammatory joint diseases. Disease activity and related systemic inflammation are the most commonly implicated non-traditional cardiovascular risk factors in inflammatory joint diseases [6,10]. In this Special Issue, Valero-Jaimes and colleagues [11] reported the baseline associations of body mass index (BMI) with disease activity in a large prospective multi-center study regarding patients with RA, AS and PsA. BMI was lowest in RA patients and highest in those with PsA [11]. In this regard, a recent mendelian randomization study documented a causal relationship between BMI and psoriasis [12]. Up to 30% of patients with psoriasis have PsA [10]. Excess adiposity can increase inflammatory joint disease activity through alterations to adipose-tissue-resident immune cell quantities and phenotypes, adipokine production and pharmacokinetics [13]. Excess adiposity can thereby reduce the response to disease-modifying agents. Valero-Jaimes and colleagues found that BMI was independently associated with disease activity in patients with RA and PsA but not in those with AS. This lack of association in AS may have been due to the more frequent use of body-weight-adjusted infliximab in patients with AS compared to participants with RA and PsA. Taken together, the adequate management of excess adiposity should be systematically targeted in patients with inflammatory joint diseases.

Angiotensin converting enzyme inhibitors (ACEis) and angiotensin II type 1 receptor blockers (ARBs) are recommended first-line agents for the treatment of hypertension in the general population, as well as in individuals with RA [10]. These agents also have anti-inflammatory effects that may benefit patients with RA [14,15]. Their use is further associated with enhanced beta cell function in patients with RA with high-grade inflammation [16]. In this Special Issue, Sluijsmans and colleagues [17] assessed the association of ACEi and ARB use with RA activity in a large retrospective cross-sectional study. ACEi and ARB use was not independently associated with RA activity, as assessed by determining the Disease Activity Score in 28 joints using C-reactive protein concentrations (DAS28-CRP). ARB use was nevertheless related to less biological disease-modifying agent use (OR (95% CI) = 1.46 (0.98–2.18)), and this was shown to have borderline significance (*p* = 0.06). Congruent with a treat-to-target treatment strategy, as applied in the clinical setting of Sluijsmans and colleagues, RA activity control was impressive, with median C-reactive protein levels and swollen and tender joint counts of 2 mg/L, 0 and 0, respectively, in both ACEi/ARB users and non-users. This may, at least in part, have explained the overall negative findings in this study. This issue merits further investigation.

The identification of carotid artery plaque via ultrasound has the potential to markedly improve cardiovascular risk assessment and management among patients with inflammatory joint disease [6]. Reported evidence indicates that this approach may in fact be most essential among RA patients from low- or middle-income populations, in whom cardiovascular risk prediction equations can be unrelated to atherosclerosis [8,18]. In a recent large 5-year prospective study [19], carotid plaque identification was most useful in predicting incident cardiovascular events and death among patients with RA. In this Special Issue, Ferraz-Amaro and colleagues [20] prospectively examined the impact of baseline disease activity on the development of carotid plaque in 160 consecutive plaque-free patients with RA during a mean (SD) period of 6 (1) years. As many as 41% of the enrolled patients with RA developed carotid plaque. This mostly occurred in older patients with RA and those with diabetes and high total and LDL cholesterol concentrations. Compared to patients with RA remission, those with moderate or high RA activity were at increased risk of developing carotid plaque, with an OR (95% CI) of 2.26 [1.02–5.00] (*p* = 0.04). In a stratified analysis, the association of RA activity with plaque development was only found in patients at low risk according to the Systematic Coronary Risk Evaluation equation. This indicates that RA activity contributes to atherosclerosis, particularly in those with a small traditional cardiovascular risk factor burden. Overall, these data emphasize the importance of not only traditional cardiovascular risk factors but also RA activity control in the prevention of atherosclerotic cardiovascular disease.

Whether disease activity can increase atherosclerosis to a similar extent in individuals with spondyloarthritis as in individuals with RA is currently uncertain. This question was addressed by Rojas-Gimenez and colleagues [21] in the present Special Issue. Ultrasound-determined carotid intima–media thickness (cIMT) and plaque were recorded in 148 patients with RA and 159 with spondyloarthritis, as well as in 40 control subjects. Disease activity was assessed using the DAS28-CRP in patients with RA and the Ankylosing Spondylitis Disease Activity Score using C-reactive protein concentrations (ASDAS-CRP) in those with spondyloarthritis. In a multivariable analysis among patient participants, RA was directly associated with cIMT but not plaque. Upon stratification by disease activity status (remission or mild versus moderate or high), an RA–cIMT relationship was found in patients who were classified in the moderate or high but not the remission or mild categories. However, disease activity status did not impact the RA–plaque relationship. Arterial plaque represents atherosclerosis more reliably than cIMT. Hence, the question of whether the impact of disease activity and related systemic inflammation on atherosclerosis is indeed larger in patients with RA compared to those with spondyloarthritis requires further study.

The issue of how to optimally assess and manage cardiovascular risk remains an evolving topic [22]. Overall, the current guidelines recommend calculating the predicted 10-year risk by using equations such as the Framingham score and Systematic Coronary Risk Evaluation (SCORE). Cardiovascular drug treatment should be initiated when the calculated risk is high or very high. However, especially in young individuals with adverse modifiable cardiovascular risk factor profiles, cardiovascular risk is often high in the long term despite a low 10-year risk. While this calls for intensive lifestyle intervention, the calculated low 10-year risk under these circumstances can make it difficult for patients to understand why control of their cardiovascular risk factors is most pertinent. Proposed strategies to assist in this prevalent context include the additional determination of 30-year and relative cardiovascular risk, as well as vascular age [22]. Vascular age calculation based on Framingham study data was reported by D’Agostino and colleagues [23]. An individual’s vascular age was defined as the age of an individual with the same predicted risk but in the absence of modifiable cardiovascular risk factors including dyslipidemia, hypertension, diabetes and smoking [23]. The determination of vascular age based on SCORE project data was also reported [24]. Another approach to determine vascular age is through the consideration of the median population composite carotid intima–media value (cIMT) [25]. In this Special Issue, Ferraz-Amaro and colleagues assessed carotid artery plaque presence and determined SCORE- and cIMT-value-based vascular ages in a large cohort of patients with RA [26]. Compared to chronological age, the mean (SD) SCORE- and cIMT-based vascular ages were increased by 4.7 (5.0) and 2.4 (17.7) years (*p* < 0.0001 for both), respectively. In receiver operator characteristic curve analysis, SCORE-based vascular ages performed just as well as SCORE in identifying carotid plaque. These findings imply that vascular age determination may assist in improving adherence to interventions that are aimed at preventing cardiovascular events in individuals with RA.

In this Special Issue, Dijkshoorn and colleagues provided a timeous updated narrative review regarding cardiovascular disease risk in individuals with RA [27]. In the absence of randomized controlled trials that evaluate the effects of antirheumatic agents on cardiovascular event rates, we ought to rely on our understanding of RA atherogenesis and observational studies, including those that originate in registry data. Oral glucocorticoid therapy at doses as low as 5 mg per day and cumulative doses of >750 mg is associated with increased cardiovascular risk in individuals with RA. In this regard, intra-articularly administered methylprednisolone likely offers advantages when used as bridge therapy upon the initiation or intensification of conventional disease-modifying agents in individuals with RA [8,16]. The use of conventional synthetic and biological disease-modifying agents, including tumor necrosis factor-α and interleukin-6 inhibitors, as well as abatacept, is associated with reduced cardiovascular risk in individuals with RA. More data are required in relation to treatment with rituximab and janus kinase inhibitors among patients with RA. Adequate cardiovascular risk stratification in individuals with RA remains a challenge. In this context and in line with the recommendations of the European League against Rheumatism [9], Dijkhoorns and colleagues recommended using a 1.5 multiplication factor upon employing cardiovascular risk equations that do not have RA included as a risk factor. An alternative and validated approach among patients with RA from high-income populations is the use of cardiovascular risk equations with low cut-off values [18]. Strikingly, and also in this regard, the Framingham score and SCORE were found to be unreliable in identifying sub-Saharan black African patients with RA with very high risks of atherosclerosis [8,18]. More recently, this phenomenon was also documented in black African individuals with chronic kidney disease [28].

As Guest Editors, we are grateful to the reviewers, and we are also grateful for the efficient and supportive organization carried out by the *JCM* team which contributed to this Special Issue. 
